# Endometriosis of the appendix presenting as acute appendicitis with unusual appearance

**DOI:** 10.1016/j.ijscr.2018.10.048

**Published:** 2018-11-03

**Authors:** B.P. St. John, A.E. Snider, H. Kellermier, S. Minhas, J.M. Nottingham

**Affiliations:** a University of South Carolina, Department of Surgery, Columbia, SC, United States; b Palmetto Health Richland, Department of Pathology, Columbia, SC, United States

**Keywords:** Endometriosis, Acute appendicitis, Microscopic findings

## Abstract

**Introduction:**

Endometriosis of the appendix is an uncommon mimicker of acute appendicitis which makes for a diagnostic dilemma.

**Presentation of case:**

We present a rare case of a menstruating woman presenting with classic symptoms of appendicitis, without the characteristic inflammatory changes seen on laparoscopy consistent with appendicitis. Instead, the appendix appeared unusually contracted on itself. Pathologic review of the appendix revealed microscopic findings of endometriosis.

**Discussion:**

We theorize the growth and shedding of the endometrial tissue during menstruation caused compression of the neural plexi in the wall of the appendix leading to the presentation mimicking acute appendicitis.

**Conclusion:**

Given the potential for endometrial appendicitis, we propose appendectomy in reproductive age female patients with right lower quadrant pain, regardless of appendix appearance on laparoscopy.

## Highlights

• Endometriosis of the appendix is an uncommon finding which can present similar to acute appendicitis.
• A key feature of the history will be ongoing menstruation in a reproductive aged female.
• We recommend removal of the appendix should be based on history and symptoms rather than appearance during laparoscopy.

## 1 Introduction

This work was reported in line with SCARE criteria [9].

Endometriosis is defined as endometrial glands and stroma in an extra-uterine site. Endometriosis is common, affecting 6–10% of women of reproductive age.^1^ However, endometriosis of the appendix is exceedingly rare with prevalence around 2.8% of women with endometriosis and 0.4% of women in the general public [2]. It is well known that endometriosis of the appendix can mimic the presentation of acute appendicitis [3]. This presentation is characterized by nausea, vomiting, abdominal pain which migrates to the right lower quadrant. Physical findings include right lower quadrant tenderness and rebound in the face of a low grade fever [4]. The treatment of acute appendicitis is currently debated between medical and surgical management between operative and antibiotic therapy [4]. This patient presented with the clinical features of acute appendicitis and she was treated with surgical management. Pathologic examination revealed endometriosis of the appendix without typical appendicitis changes.

## 2 Case presentation

A 29-year-old healthy African American female presented to the emergency department complaining of a 1-day history of peri-umbilical pain migrating to the right lower abdominal quadrant with associated anorexia, nausea, and vomiting. She had an onset of menses the day prior to onset of abdominal pain. On physical exam, the abdomen was soft, non-distended, but tender to palpation over McBurney’s point. Vital signs were within normal limits without notable fever or tachycardia. Blood work revealed an elevated white blood count of 17.4 K/UL. Alvarado score was calculated to be 9. CT of the abdomen with IV contrast exhibited no evidence of acute intra-abdominal or intra-pelvic process. Ultrasound of the pelvis disclosed dilated non-compressible distal appendix suggestive of appendicitis.

Diagnostic laparoscopy was performed which found 30 cc of blood in the pelvis attributed to a ruptured 3 cm left hemorrhagic ovarian cyst. The appendix appeared unusually contracted upon itself without evidence of erythema or surrounding acute inflammation. No peritoneal studding or endometrial implants were identified on laparoscopic evaluation of the abdomen or pelvis, and the omentum was not found in the right lower quadrant. She recovered uneventfully from her operation, and in follow-up her pre-operative pain had disappeared.

**Fig. 1:**
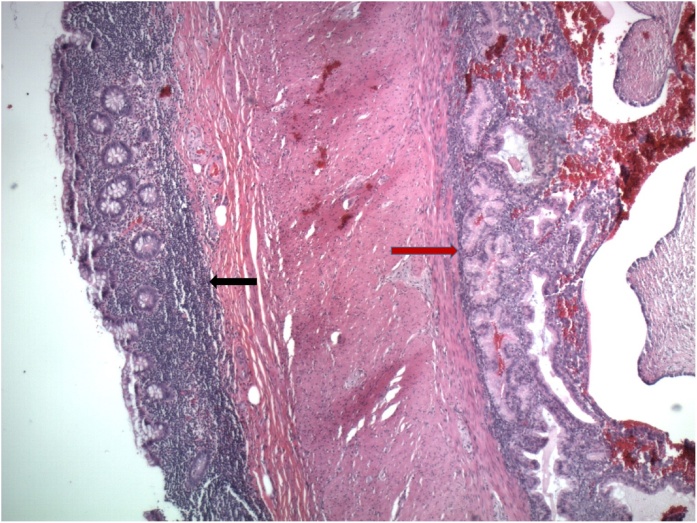
H&E, 40× magnification.

**Fig. 2:**
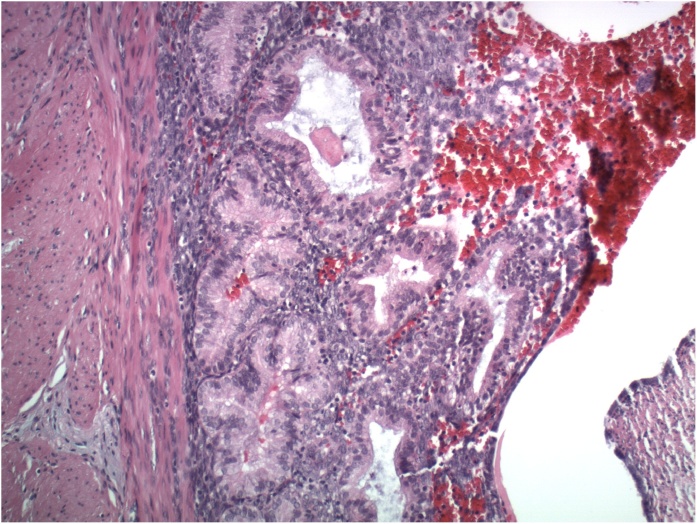
H&E, 100× magnification.

Microscopic examination of the appendix showed no pathologic evidence for acute appendicitis. The appendiceal lumen was lined by normal-appearing appendiceal mucosa (Fig. 1), and the serosa showed no polymorphonuclear cells but did show collections of benign endometrial-type glands and stroma, consistent with endometriosis (Red arrow, right). (H&E, 40× magnification) In Fig. 2, higher power view showed benign endometrial-type glands and stroma. (H&E 100× magnification)

## 3 Discussion

The commonalities between the presentation of this case and that of a classic case of acute appendicitis include: 1) abdominal pain beginning in the periumbilical region followed by migration to the right lower quadrant 2) nausea and vomiting 3) leukocytosis. However, a key component of the history was the fact that this patient was menstruating. Multiple case reports have described the involvement of menstruation in the presentation of endometriosis of the appendix [5,6]. This key element of the history should have prompted the surgeon to look not only for signs of acute appendicitis but also for signs of endometriosis during laparoscopy. The surgeon should also examine the uterus, tubes and ovaries for a complete evaluation for endometriosis and other gynecologic pathology. The signs of endometriosis being numerous adhesions with red, white or black nodules in the peritoneal cavity and pelvis [7].

This case did not have the classic macroscopic findings of either acute appendicitis or endometriosis. Instead, the appendix appeared to have been unusually contracted upon itself. Fifteen to thirty percent of appendices removed for symptoms of appendicitis are grossly normal and there is question to the efficacy of removing the appendix in this scenario [8]. This case supports the practice of removing the appendix in women of reproductive age with symptoms of acute appendicitis in the setting of menstruation and no characteristic macroscopic findings on laparoscopy. Pathology should be considered the gold standard for assessment of appendiceal processes.

There are currently three leading theories for the pathogenesis of endometriosis: retrograde menstruation with implantation and failure of immunologic clearance, coelomic metaplasia, and hematologic or lymphatic metastasis [1]. This case supports retrograde menstruation as the cause due to the endometrial tissue involvement on the outermost layer of the appendix. We theorize that the symptoms are not caused by a true luminal obstruction of the appendix, but rather due to compression of neural plexi. The expansion and growth of the endometrial tissue into the layers of the appendix, during menstruation, caused compression of the neural plexi located in the wall of the appendix leading to visceral pain, nausea, vomiting and anorexia. Therefore, the problem is not due to inter-luminal pressure but rather due to invasion and compression of tissue by lymphoid nodules, abscesses or endometriosis.

## 4 Conclusion

This case details a female of reproductive age presenting with signs of acute appendicitis during menstruation with subsequent findings of endometriosis on pathology. This supports the need for laparoscopic examination and appendectomy even if there are no characteristic gross abnormalities identified. Surgical management with pathologic evaluation merits designation as the gold standard for treating and identifying appendiceal endometriosis.

## Conflicts of interest

None.

## Funding source

No sources of funding were obtained for this project.

## Ethical approval

This study was exempt from ethical approval at our institution as it was an observational finding in regular practice.

## Consent

Written informed consent was obtained from the patient for publication of this case report and accompanying images. A copy of the written consent is available for review by the Editor-in-Chief of this journal on request.

## Author contribution

Dr. James Nottingham and Dr. Alicia Snider performed the operation. Dr. Harry Kellermier performed the pathology read and interpretation. Bradley St. John wrote and formatted the original manuscript. Dr. Snider and Dr. Nottingham reviewed and edited the manuscript. Shubhanjali Minhas produced the images and formatted the original manuscript.

## Registration of research studies

This case report does not qualify as research as it was an observational finding on routine procedure

## Guarantor

Dr. Alicia Snider, Dr. James Nottingham, and Bradley St. John

## Provenance and peer review

Not commissioned, externally peer reviewed.
